# Supplementary material for: Identification of Factors Affecting Environmental Contamination Represented by Post-Hatching Eggshells of a Common Colonial Waterbird with Usage of Artificial Neural Networks
Source: Sensors (Basel). 2022 May 13;22(10):3723. doi: 10.3390/s22103723 (PMC9143455; doi:10.3390/s22103723)
Supplement: Supplementary file 1 [file sensors-22-03723-s001.zip › sensors-1700562-supplementary.pdf]

Supplementary material

Colony codes: *GK – Gardzka Kępa (12), KR – Kąty Rybackie (12), BR – Brwilno near Płock (12), CH – Chutkowice (12), KS – Kiersity (12), GA – Gaładuś (12), GO – Gołdap (12), OS – Osiek (10), JS – Sławskie Lake (12), MO – Mosty (11), ZB – Zborowskie Lake (12), DD – Dzierżno Duże Lake (12), JA – Jawory (12), PO – Pogorzałki (10), MA – Malczyce (12), LI – Limajno Lake (12), KI – Kiełcz (12), RA – Raszyn (12), OT – Otmuchów (12), ST – Stobrawa (12), PL – Płoskinia (12), WR – Wrocław Zoo (4).*

Descriptions: ARA_LAND - Arable land, ARTIF_AREAS - Artificial, non-agricultural vegetated areas, FORESTS- Forests, HET_AGRO - Heterogeneous agricultural areas, INDUST_UNITS- Industrial, commercial and transport units, INL WAT - Inland waters, INL_WET - Inland wetlands, MAR_WAT- Marine waters, MINE_DUMP - Mines, dump and construction sites, OPEN_SPACE - Open spaces with little or no vegetation, PASTURES – Pastures, PERM_CROPS - Permanent crops, SCRUB_VEGET - Scrub and/or herbaceous vegetation associations, URBAN FABRIC – Urban fabric

Table S1. Proportion of particular habitat types in a 20 km buffers around the studied colonies of grey herons. Habitat types according to the Corine Land Cover CLC2012 model.

Code	ARA_LAND	ARTIF_AREAS	FORESTS	HET_AGRO	INDUST_UNITS	INL WAT	INL_WET	MAR_WAT	MINE_DUMP	OPEN_SPACE	PASTURES	PERM_CROPS	SCRUB_VEGET	URBAN_FABRIC
GK	0.257775	0.001202	0.308387	0.033062	0.002538	0.010272	0.018111	0.195427	0.000001	0.000186	0.14477	0.000201	0.006139	0.02193
KR	0.288168	0.000295	0.051412	0.026879	0.000001	0.013441	0.007434	0.578013	0.000001	0.00247	0.010629	0.001064	0.00053	0.019667
BR	0.450062	0.003617	0.272359	0.107853	0.013146	0.052205	0.00324	0.000001	0.000483	0.00102	0.027995	0.000517	0.034797	0.032707
CH	0.592998	0.000001	0.191245	0.080271	0.000001	0.006997	0.000276	0.000001	0.000001	0.00022	0.083937	0.001765	0.006117	0.036173
KS	0.648159	0.001472	0.136618	0.064268	0.001433	0.024771	0.012249	0.000001	0.005164	0.000001	0.077544	0.000421	0.008868	0.019033
GA	0.343597	0.000223	0.202156	0.257713	0.00048	0.040344	0.003279	0.000001	0.000001	0.000001	0.124235	0.000431	0.014869	0.012671
GO	0.394929	0.000001	0.346778	0.144692	0.001392	0.014256	0.003463	0.000001	0.000001	0.000001	0.060481	0.000001	0.025419	0.008588
OS	0.346489	0.000947	0.458289	0.024796	0.000001	0.025101	0.000748	0.000001	0.000231	0.000001	0.099326	0.001343	0.022958	0.019773
JS	0.338262	0.001800	0.482496	0.010593	0.001075	0.019955	0.002508	0.000001	0.000475	0.000001	0.094882	0.000001	0.010542	0.037408
MO	0.110827	0.005996	0.232592	0.029777	0.014701	0.001142	0.004073	0.484395	0.000605	0.003922	0.039351	0.000001	0.001153	0.071465
ZB	0.343782	0.000358	0.480979	0.046024	0.003126	0.005743	0.001025	0.000001	0.000973	0.000001	0.065841	0.000001	0.017365	0.034784
DD	0.426361	0.008946	0.318893	0.025836	0.031172	0.008537	0.000001	0.000001	0.006565	0.000001	0.039201	0.000001	0.048201	0.086287
JA	0.349504	0.000001	0.498988	0.034097	0.000001	0.014637	0.000305	0.000001	0.000001	0.000001	0.054046	0.000001	0.026854	0.021567
PO	0.291319	0.004051	0.282753	0.07966	0.005775	0.005249	0.035403	0.000001	0.001933	0.000001	0.229229	0.000205	0.007508	0.056915
MA	0.612809	0.003586	0.199514	0.034116	0.004476	0.010049	0.000208	0.000001	0.002005	0.000001	0.046808	0.001126	0.028146	0.057159
LI	0.463806	0.002574	0.30875	0.057951	0.006636	0.022811	0.003158	0.000001	0.000001	0.000001	0.071088	0.000001	0.030257	0.032969
KI	0.430211	0.002525	0.411499	0.035999	0.004432	0.007315	0.00128	0.000001	0.000588	0.000001	0.050219	0.001568	0.013958	0.040405
RA	0.27574	0.046278	0.139279	0.086611	0.054652	0.013937	0.000001	0.000001	0.008095	0.001513	0.060199	0.012319	0.013571	0.287806
OT	0.594428	0.004652	0.16694	0.071111	0.003291	0.026494	0.004947	0.000001	0.001672	0.000001	0.067686	0.000001	0.005873	0.052906
ST	0.52346	0.002325	0.308152	0.011286	0.005896	0.015746	0.000001	0.000001	0.002794	0.000001	0.067916	0.00128	0.012988	0.048159
PL	0.586828	0.000874	0.284584	0.044194	0.002612	0.002845	0.00651	0.009612	0.000001	0.000001	0.026838	0.000001	0.020151	0.01495
WR	0.542616	0.032264	0.117008	0.026828	0.037269	0.010384	0.000001	0.000001	0.004568	0.000001	0.071213	0.001857	0.009283	0.14671

Table S2. Concentration of particular elements in post-hatching eggshells of grey herons in the studied colonies.

Element	Al	As	Cd	Cr
Code	mean (range) [mg/kg]	mean (range) [mg/kg]	mean (range) [μg/kg]	mean (range) [mg/kg]
GK	3.80 (3.05-5.85)	0.28 (0-0.52)	6.6 (3.5-18.5)	0.11 (0.02.-0.23)
KR	16.09 (4.41-36.31)	0.31 (0-0.60)	14.9 (8.3-21.1)	0.51 (0.41-0.65)
BR	12.31 (5.06-16.92)	0.39 (0.18-0.73)	14.1 (9.1-19.6)	0.46 (0.34-0.69)
CH	13.97 (10.46-17.45)	0.30 (0.06-0.51)	12.7 (6.5-23.2)	0.45 (0.31-0.74)
KS	13.33 (3.29-17.10)	0.46 (0.23-0.65)	10.4 (4.5-18.6)	0.33 (0.07-0.49)
GA	13.31 (9.78-15.60)	0.36 (0.15-0.51)	6.4 (0-12.8)	0.02 (0-0.05)
GO	18.11 (14.09-35.58)	0.44 (0-1.01)	6.9 (2.9-14.6)	0.04 (0-0.15)
OS	12.28 (9.74-15.50)	0.31 (0-0.78)	7.2 (0-0.0140)	0.03 (0-0.12)
JS	10.38 (2.56-28.70)	0.39 (0.22-1.26)	7.3 (0-16.2)	0.03 (0-0.13)
MO	12.66 (8.92-27.43)	0.53 (0-1.32)	8.1 (2.5-14.9)	0.09 (0-0.24)
ZB	9.13 (4.87-19.58)	0.48 (0.26-0.78)	10.7 (0-29.3)	0.04 (0-0.11)
DD	8.37 (3.17-17.91)	0.38 (0.17-0.48)	5.2 (0-7.9)	0.08 (0-0.43)
JA	10.87 (7.73-27.40)	0.89 (0.54-1.13)	37.5 (28.4-48.7)	0.06 (0-0.19)
PO	6.18 (5.36-7.31)	0.75 (0.33-1.28)	43.7 (34.9-56.3)	0.16 (0-0.35)
MA	16.02 (8.72-28.99)	1.07 (0.45-1.37)	43.6 (33.9-57.7)	0.11 (0-0.27)
LI	8.27 (5.00-17.51)	0.77 (0-1.08)	29.9 (11.1-46.1)	0.25 (0-0.39)
KI	12.02 (7.51-17.51)	0.46 (0.23-0.64)	37.2 (28.8-46.3)	0.09 (0-0.18)
RA	6.79 (5.19-11.08)	0.63 (0-0.94)	22.8 (9.8-37.5)	0.09 (0-0.23)
OT	13.45 (5.57-33.13)	0.70 (0-1.29)	32.3 (22.2-42.2)	0.08 (0-0.22)
ST	11.18 (5.55-43.17)	1.00 (0.59-1.40)	36.0 (22.1-50.4)	0.05 (0-0.18)
PL	13.83 (5.82-61.55)	0.51 (0-1.26)	25.3 (15.7-35.9)	0.07 (0-0.19)
WR	14.34 (5.99-32.89)	0.91 (0.53-1.20)	27.7 (22.6-31.1)	0.06 (0-0.21)
	Cu	Fe	Hg	Mn
	mean (range) [mg/kg]	mean (range) [mg/kg]	mean (range) [mg/kg]	mean (range) [mg/kg]
GK	1.51 (0.80-1.91)	5.11 (2.75-10.64)	0.08 (0.02-0.15)	1.12 (0.46-1.94)
KR	2.90 (2.31-3.49)	9.98 (4.74-26.41)	0.15 (0-0.71)	1.60 (0.75-2.68)
BR	2.74 (2.33-3.69)	8.34(6.05-10.08)	0.12 (0.04-0.19)	1.74 (0.91-2.76)
CH	2.35 (2.05-3.09)	9.02 (6.91-11.66)	0.13 (0.03-0.16)	2.72 (1.49-5.04)
KS	2.14 (0-2.48)	6.36 (4.70-7.76)	0.09 (0.05-0.14)	1.97 (0.67-4.72)
GA	2.04 (1.07-5.61)	5.84 (3.16-13.44)	0.11 (0-0.17)	1.14 (0.55-2.12)
GO	1.51 (1.18-1.79)	4.64 (2.42-9.34)	0.09 (0.05-0.16)	2.13 (0.41-2.79)
OS	1.42 (0.85-1.99)	3.90 (2.44-6.71)	0.09 (0-0.17)	0.72 (0.33-1.28)
JS	1.45 (0.91-1.79)	3.25 (1.76-4.89)	0.10 (0.03-0.17)	1.71 (0.43-3.94)
MO	1.62 (1.17-2.12)	18.89 (2.15-85.26)	0.08 (0.03-0.13)	4.46 (0.53-17.14)
ZB	1.75 (1.21-2.06)	8.27 (4.24-17.06)	0.12 (0.05-0.23)	4.17 (0.36-27.28)
DD	1.20 (0.79-1.53)	8.20 (4.07-19.27)	0.09 (0.06-0.14)	1.34 (0.61-3.61)
JA	1.67 (1.19-1.98)	10.65 (6.45-31.19)	0.29 (0.09-0.82)	5.59 (1.69-13.25)
PO	1.89 (1.68-2.25)	6.67 (5.05-8.68)	0.24 (0.13-0.31)	0.59 (0.35-1.31)
MA	1.33 (0.95-1.69)	19.37 (8.85-38.82)	0.22 (0.12-0.32)	1.92 (0.85-5.69)
LI	1.18 (0.73-1.41)	7.52 (4.15-10.91)	0.30 (0.14-0.74)	1.49 (0.78-2.59)
KI	1.52 (1.28-1.71)	14.71 (7.29-31.89)	0.25 (0.14-0.33)	4.89 (1.65-17.56)
RA	1.11 (0.74-1.58)	8.51 (6.11-13.66)	0.20 (0.09-0.34)	4.29 (0.96-16.17)
OT	1.49 (0.88-1.78)	12.35 (5.67-38.71)	0.26 (0.13-0.37)	6.04 (1.01-11.26)
ST	1.22 (0-1.85)	9.46 (5.78-16.69)	0.23 (0.13-0.29)	3.63 (0.83-7.13)
PL	1.67 (1.21-2.02)	5.04 (2.97-7.95)	0.16 (0.08-0.21)	1.98 (0.93-3.42)
WR	1.64 (1.48-1.93)	5.35 (4.77-6.41)	0.17 (0.11-0.24)	0.77 (0.38-1.01)

Table S2. *Count.*

Element	Mo	Ni	Pb	Sc
Code	mean (range) [mg/kg]	mean (range) [mg/kg]	mean (range) [mg/kg]	mean (range) [mg/kg]
GK	0.032 (0-0.099)	0.12 (0.04-0.25)	0.57 (0.39-0.77)	0.005 (0-0.019)
KR	0.092 (0.042 - 0.189)	0.18 (0.03-0.27)	0.66 (0.36-0.93)	0.019 (0-0.044)
BR	0.069 (0.021-0.120)	0.19 (0.06-0.28)	0.55 (0.45-0.75)	0.014 (0.005-0.027)
CH	0.064 (0.024-0.133)	0.22 (0.14-0.31)	0.46 (0.12-0.89)	0.016 (0.006-0.032)
KS	0.045 (0-0.081)	0.19 (0.05-0.27)	0.47 (0.25-0.68)	0.021 (0-0.045)
GA	0.010 (0-0.052)	0.14 (0.10-0.21)	0.32 (0.05-0.63)	0.014 (0-0.030)
GO	0.010 (0-0.027)	0.14 (0.07-0.18)	0.51 (0.37-0.69)	0.019 (0-0.039)
OS	0.010 (0-0.029)	0.12 (0.09-0.16)	0.51 (0.25-0.76)	0.014 (0-0.026)
JS	0.015 (0-0.046)	0.0935 (0-0.1481)	0.42 (0.26-0.57)	0.005 (0-0.019)
MO	0.008 (0-0.042)	0.130 (0.095-0.174)	0.48 (0.25-0.60)	0.007 (0-0.018)
ZB	0.011 (0-0.036)	0.067 (0-0.232)	0.49 (0.27-1.34)	0.003 (0-0.011)
DD	0.015 (0-0.051)	0.0142 (0-0.0776)	0.53 (0.28-0.83)	0.004 (0-0.021)
JA	0.043 (0-0.212)	0.162 (0.122-0.186)	0.534 (0.085-1.106)	0.097 (0.042-0.168)
PO	0.024 (0-0.060)	0.152 (0.137-0.177)	0.448 (0.088-0.626)	0.037 (0-0.085)
MA	0.029 (0-0.117)	0.137 (0.101-0.184)	0.398 (0.124-0.908)	0.077 (0.034-0.164)
LI	0.023 (0-0.190)	0.130 (0.111-0.155)	0.457 (0.096-0.851)	0.055 (0.021-0.104)
KI	0.005 (0-0.058)	0.135 (0.111-0.159)	0.634 (0.232-1.087)	0.110 (0-0.186)
RA	0.008 (0-0.097)	0.129 (0.111-0.172)	0.531 (0.231-0.883)	0.080 (0.013-0.165)
OT	0.019 (0-0.072)	0.115 (0.094-0.160)	0.416 (0.125-0.723)	0.067 (0-0.200)
ST	0.018 (0-0.086)	0.121 (0.091-0.157)	0.372 (0-0.657)	0.081 (0.035-0.157)
PL	0.014 (0-0.049)	0.122 (0.103-0.151)	0.366 (0.037-0.801)	0.044 (0.025-0.072)
WR	0.018 (0-0.052)	0.122 (0.106-0.138)	0.415 (0.228-0.538)	0.056 (0.035-0.075)
	Se	Sr	V	Zn
	mean (range) [ppm]	mean (range) [ppm]	mean (range) [μg/kg]	mean (range) [mg/kg]
GK	0.577 (0.186-1.121)	151.17 (81.74-203.68)	55.5 (0-142.0)	3.39 (1.21-6.63)
KR	0.927 (0.193-2.508)	186.28 (94.77-265.19)	26.0 (0-88.1)	7.74 (5.14-10.49)
BR	0.493 (0.243-0.884)	85.16 (55.69-112.26)	33.4 (0-168.5)	9.15 (6.12-16.86)
CH	0.484 (0.037-0.938)	60.36 (26.37-83.65)	28.2 (0-89.8)	9.59 (6.36-18.12)
KS	0.573 (0.132-0.959)	75.35 (42.46-129.62)	21.9 (0-144.0)	5.63 (2.74-7.94)
GA	0.441 (0.204-0.801)	47.61 (26.36-95.19)	23.8 (0-95.1)	8.49 (0.09-15.28)
GO	0.539 (0.252-0.936)	41.76 (31.39-60.10)	25.6 (0-94.3)	7.36 (1.86-14.62)
OS	0.565 (0-0.793)	83.87 (31.59-125.59)	69.4 (0-158.5)	5.63 (2.83-10.98)
JS	0.5151 (0.2126-0.9887)	60.21 (43.85-86.45)	32.0 (0-122.6)	4.18 (1.77-7.26)
MO	0.552 (0.315-0.876)	232.90 (152.10-336.48)	50.8 (0-126.9)	5.50 (2.37-12.02)
ZB	0.614 (0.314-0.966)	136.39 (68.43-207.66)	50.5 (0-123.5)	12.21 (7.98-20.75)
DD	0.6451 (0.1616-0.9659)	193.46 (138.60-223.18)	33.1 (0-87.9)	5.02 (2.93-7.81)
JA	0.601 (0.259-1.063)	72.94 (41.77-93.45)	13.32 (0-358.9)	26.12 (20.03-45.04)
PO	0.526 (0.032-1.041)	45.55 (28.97-64.75)	159.2 (96.6-258.2)	6.99 (2.19-14.47)
MA	0.716 (0.2908-1.4359)	60.69 (38.86-83.71)	163.9 (13.3-298.5)	5.55 (3.29-9.05)
LI	1.386 (0-3.696)	55.59 (32.76-91.93)	5.4 (0-202.6)	24.96 (13.62-35.07)
KI	2.163 (1.518-2.589)	60.44 (30.87-80.41)	169.9 (37.2-283.9)	20.93 (7.85-33.49)
RA	2.094 (1.458-2.719)	50.69 (36.46-78.37)	94.3 (0-248.3)	20.07 (13.33-31.81)
OT	1.960 (1.426-2.410)	69.57 (38.69-120.03)	133.9 (0-267.0)	6.23 (3.37-9.98)
ST	1.9251 (1.2553-2.7669)	70.46 (48.65-93.94)	140.5 (15.2-269.3)	12.83 (4.26-26.88)
PL	1.8099 (1.0937-2.4916)	83.90 (58.59-146.86)	116.9 (0-283.5)	5.25 (2.08-9.42)
WR	2.148 (1.810-2.612)	63.35 (42.006-85.087)	210.3 (158.1-243.8)	13.09 (5.82-16.25)

Concentrations of Ca and Mg – main components of an eggshell ranged between 334.11-370.26 g/kg and 0.89-1.27 g/kg, respectively.
